# Complete Genome Sequences of Four Soil-Derived Isolates for Studying Synthetic Bacterial Community Assembly

**DOI:** 10.1128/MRA.00848-21

**Published:** 2021-11-18

**Authors:** Carlos N. Lozano-Andrade, Mikael Lenz Strube, Ákos T. Kovács

**Affiliations:** a Bacterial Interactions and Evolution Group, DTU Bioengineering, Technical University of Denmark, Kongens Lyngby, Denmark; b Bacterial Ecophysiology and Biotechnology Group, DTU Bioengineering, Technical University of Denmark, Kongens Lyngby, Denmark; SIPBS, University of Strathclyde

## Abstract

Here, we report the complete genome sequences of four bacterial soil isolates, *Chryseobacterium* sp., Stenotrophomonas indicatrix, *Pedobacter* sp., and Rhodococcus globerulus. These isolates can be used for studying microbial interactions and community assembly *in vitro*.

## ANNOUNCEMENT

Soil microbes play diverse and often pivotal roles in ecosystem services, driving biogeochemical cycles, plant growth, and life above and below ground (1, 2).

These activities are performed by complex communities composed of several interacting species, rather than single species. The high complexity of soil microbial communities poses great difficulty for experimentally testing ecological hypotheses, such as microbe-plant interactions or the underlying mechanisms of community assembly. Therefore, experimentally tractable and manipulatable synthetic communities are needed as a model for addressing fundamental questions in microbial ecology (3). We experimented with a four-member synthetic bacterial community to study its assembly and functionality. The isolates were obtained from 1 g soil sample (Dyrehaven, Denmark; coordinates, 55.788800, 12.558300), using serial dilutions plated in 0.1× TSA. Here, we announce the complete genome sequences of *Chryseobacterium* sp. strain D764, Stenotrophomonas indicatrix D763, *Pedobacter* sp. strain D749, and Rhodococcus globerulus D757.

For genome sequencing, the strains were grown overnight in LB at 24°C, and genomic DNA (gDNA) was extracted using the GeneMATRIX bacterial and yeast genomic DNA purification kit (EURx, Gdansk, Poland). Two separate DNA extractions were conducted for each sequencing technology, yielding at least 1 μg of gDNA, quantified using Qubit. For Illumina sequencing, a library was prepared using the NEBNext DNA library kit (New England BioLabs, USA).The gDNA was randomly fragmented to 350 bp, end polished, A-tailed, ligated with adapters, and PCR enriched. Then, paired-end reads were generated on the NovaSeq 600 platform with 2 × 150-bp reads. For Nanopore sequencing, a ligation sequencing kit (SQK-LSK109) was used with the native barcoding expansion 1-12 kit (EXP-NBD104), following the manufacturer’s instructions. The libraries were sequenced using an R9.4.1 flow cell on a MinION device running a 48-h sequencing cycle. The reads were base called and demultiplexed using Guppy v.3.1.5 (ONT).

For *de novo* assembly, the Illumina and Nanopore reads were adapter and quality trimmed using AdapterRemoval v.2.3.1 (4) and Porechop v.0.2.4 (5), respectively. Subsequently, the trimmed reads from both platforms were hybrid assembled using Unicycler v.0.4.8 (6). The complete, circular, and rotated chromosome of each strain produced using Unicycler was evaluated using Bandage v.0.8.1 (7) and BUSCO v.4.1.4 (8) to evaluate the core gene content and CheckM v.1.0.8 for the completeness and contamination levels (9). The chromosomes were annotated using the NCBI Prokaryotic Genome Annotation Pipeline. The species phylogeny was analyzed using autoMLST (10) and TYGS (11). Default parameters were used for all software. *Pedobacter* sp. D749 and *Chryseobacterium* sp. D764 had <95% average nucleotide identity (ANI) compared to the genomes of the type strains and thus could represent novel species.

### Data availability.

The raw sequencing data have been deposited at the NCBI Sequence Read Archive under accession number PRJNA743326 (SRX11393888 and SRX11393892 for strain D749, SRX11393889 and SRX11393893 for strain D757, SRX11393890 and SRX11393894 for strain D763, and SRX11393891 and SRX11393895 for strain D764 for the Illumina and Nanopore reads, respectively), and the genome assemblies have been deposited in GenBank under BioProject accession number PRJNA743326. Detailed information for each strain is listed in Table 1.

**TABLE 1:** Strain names, accession numbers, and genome characteristics of the soil isolates used in this study

Strain^*a*^	GenBank accession no.	No. of assembled reads		Genome assembly size (bp)	Avg read length for Nanopore reads (bp)	Maximum read length for Nanopore reads (bp)	G+C content (%)	No. of CDS^*b*^	No. of rRNAs	No. of tRNAs	Completeness (%)^*c*^	Contamination (%)^*c*^	Complete BUSCO core genes (%)^*d*^	Topology
		Illumina	Nanopore											
*Pedobacter* sp. D749	CP079218.1	3,337,001	8,681	5,843,246	10,467.7	84,368	38.4	4,895	15	54	98.09	0.19	98.5	Circular
Rhodococcus globerulus D757	CP079698.1	4,662,334	7,757	6,739,623	6,215.7	28,384	61.7	6,091	15	52	99.56	0.89	99	Circular
Stenotrophomonas indicatrix D763	CP079106.1	4,015,635	7,238	4,615,841	13,210.5	78,776	66.3	4,108	13	70	100	0.09	99.9	Circular
*Chryseobacterium* sp. D764	CP079219.1	3,464,854	7,638	4,921,682	21,343.3	92,855	36.2	4,343	18	85	100	0.25	97.4	Circular

a Species delineation was performed using TYGS and autoMLST. For TYGS, the digital DNA-DNA hybridization (dDDH) threshold value was >70%.

b CDS, coding DNA sequences.

c Estimated using CheckM v.1.0.8.

d Complete and single-copy benchmarking universal single-copy orthologs (BUSCOs).
